# Accurate method for evaluating the duration of the entire radiotherapy process

**DOI:** 10.1002/acm2.12959

**Published:** 2020-07-25

**Authors:** Chenlei Guo, Peng Huang, Yexiong Li, Jianrong Dai

**Affiliations:** ^1^ Department of Radiation Oncology National Cancer Center/National Clinical Research Center for Cancer/Cancer Hospital Chinese Academy of Medical Sciences and Peking Union Medical College Beijing China

**Keywords:** MOSAIQ, radiotherapy, time evaluation, workflow management

## Abstract

**Background and purpose:**

Along with the increasing demand for high‐quality radiotherapy and the growing number of high‐precision radiotherapy devices, precise radiotherapy workflow management and accurate time evaluation of the entire radiotherapy process are crucial to providing appropriate, timely treatment for cancer patients. This study therefore aimed to establish an accurate, reliable method for evaluating the duration of the radiotherapy process, from beginning to end, based on real‐time measurement data. These data are vital for improving the quality and efficiency of radiotherapy delivery.

**Materials and methods:**

Altogether, 17 620 cancer patients’ radiotherapy experiences were measured in real time in our radiation oncology department. The process was divided into five sequential core modules, with the start and stop times of each module automatically recorded using MOSAIQ software, an automated radiotherapy management system. The duration for each module and the total duration of the entire process were then automatically calculated and qualitatively analyzed.

**Results:**

The analysis showed significant treatment–time differences depending on the tumor site, which provided a practical reference for improvement of previous treatment modules and appointments management. In all, >60% of the cancer patients’ total treatment time could be shortened.

**Conclusions:**

We established a reliable method for evaluating the overall duration of radiotherapy protocols. The results pointed out a clear pathway by which we could improve future radiotherapy workflow management and appointment systems.

## 1 INTRODUCTION

Although radiotherapy has long been recognized as an effective, relatively inexpensive modality for treating cancer, it has undergone major technological development in recent decades.^1^, ^2^, ^3^, ^4^ Radiotherapy requires the collaboration of several medical specialties, including physicians, medical physicists, technicians/therapists, and nurses. Along with the increasing demand for high‐quality radiotherapy and the growing number of high‐precision treatment devices, the entire radiotherapy process itself inevitably becomes more complex, with the result that management of the radiotherapy workflow — for example, appointment conflicts, unnecessary work — is outdated, resulting in prolonged patients’ waiting time and delayed treatment. Such conditions increase patients’ anxiety and, more importantly, allow tumor progression. The existing radiotherapy workflow therefore needs to be optimized, which requires accurate data collection regarding the entire radiotherapy process for use as a reference.^5^, ^6^, ^7^

Several relevant international organizations have offered recommendations and guidelines for accurately assessing the time data of radiotherapy, which have improved the management of radiotherapy workflow.^4^, ^5^, ^8^, ^9^, ^10^, ^11^, ^12^, ^13^, ^14^, ^15^. Nevertheless, only a few studies focused on analyzing data based on real‐time statistics. A series of studies published in recent years by the German Society of Radiation Oncology (DEGRO)^8^, ^16^, ^17^, ^18^, ^19^, ^20^, ^21^, ^22^, ^23^, ^24^ might be the most systematic, comprehensive reports in the relevant literature. The DEGRO trials yielded eligible time measurement data for several core radiotherapy modules — for example, preparation for RT; RT planning; performance of RT; completion/follow‐up appointment — which served as reference guides for actual radiotherapy. DEGRO, however, analyzed only statistical data based on the manually recorded working time of the core modules, which might lead to great uncertainties and errors as it failed to cover all the time spent during the entire radiotherapy process, including the preparation and waiting time between modules. Clearly, it is of vital importance to evaluate all the time spent for the entire radiotherapy process.

To overcome the abovementioned shortcomings, this study aimed to establish an accurate, reliable method for evaluating the duration of the entire radiotherapy process. In our department, all physicians treating NKT, NPC, CNS, and brain metastases are divided into one treatment group, referred to as the “head and neck (H&N)” group. All physicians treating lung, esophagus, breast, and lymph node are divided into the second treatment group, referred to as the “Thorax/breast” group. And all physicians treating rectum, liver, prostate, gynecologic, extremities, and among others are divided into the third treatment group, referred to as the “Abdomen/Pelvis/Extremity” group. Thus, the statistical data of the actual time spent during the entire radiotherapy process for three treatment groups were recorded in real time and analyzed in an automated management system.

The study then sought to determine opportunities for improving the total time consumed to complete the entire clinical radiotherapy process.

## 2 MATERIALS AND METHODS

**Fig. 1:**
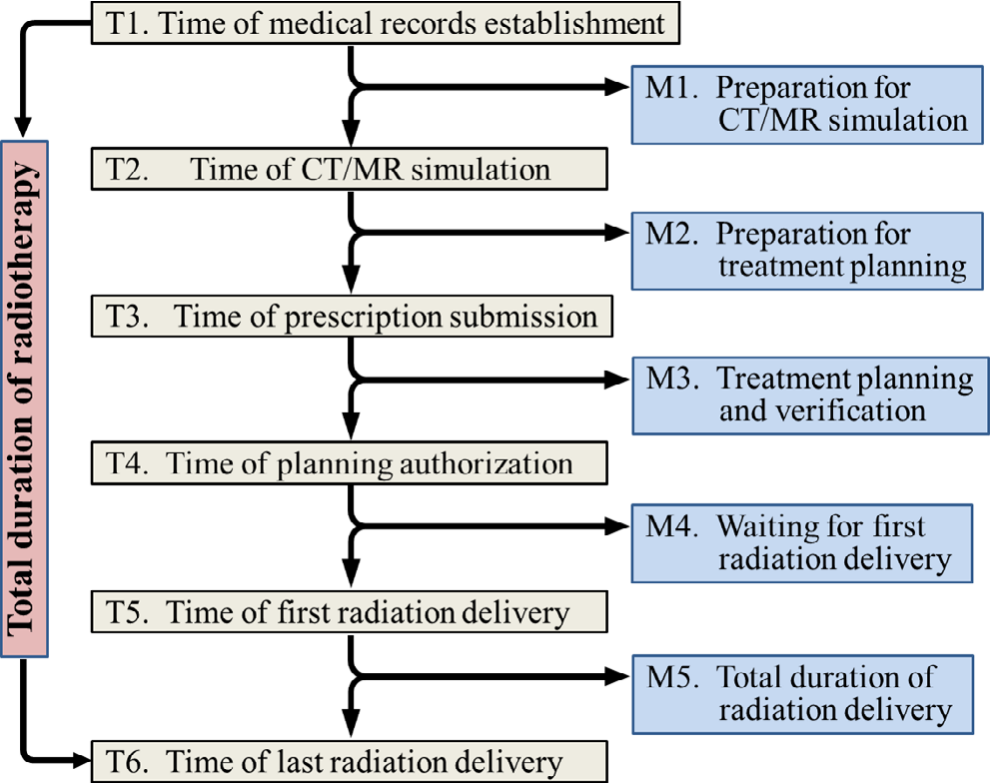
Overview of entire process of radiotherapy.

The study statistically analyzed time information for 17 620 radiotherapy patients (5125 H&N tumors, 6594 thoracic tumors, and 5901 abdominal tumors) in our department from January 2016 to December 2018. To cover the entire process of conventional radiotherapy workflow,^8^, ^22^ six critical time points (Fig. 1, T1–T6) were manually selected and automatically recorded in an integrated radiotherapy information management system, MOSAIQ (Elekta, Stockholm, Sweden).^25^ These time points were named the time of medical records establishment, time of CT/MR simulation, time of prescription submission, time of planning authorization, time of first radiation delivery, and time of last radiation delivery. Thus, the entire radiotherapy process was divided into five sequential modules (Fig. 1, M1–M5) called the preparation for CT/MR simulation, preparation for treatment planning, treatment planning and verification, waiting for first radiation delivery, and total duration of radiation delivery.

When a new module starts, the responsible physicians, medical physicists, or technicians/therapists first manually set the module’s status on Mosaiq to “Schedule,” and when work is done, they set the status to “Complete,” and the status of the next module will be automatically switched to the “Prepare,” to remind next group to start clinic work. The status (“Prepare,” “Schedule,” and “Complete”) setting time for each patient was automatically recording in the Mosaiq system and time information were extracted from the Mosaiq database, using the commercial software ‘Crystal Reports’ (SAP AG 2010, version: 14.0.2.364 RTM). Thus, the time duration of each module was obtained by noting the time consumed between the “Schedule” and “Complete” time points, and the waiting time in between two modules was obtained by “Complete” and “Prepare” time points. Clearly, the total duration of the radiotherapy process was obtained by noting the time consumed between the overall first and last time points — the Total duration of radiotherapy.

The mean, median, 5% and 95% percentiles, and 25% and 75% quartiles of all six time durations for treating three tumor sites were calculated. The nonparametric Mann–Whitney *U* test module of standard statistics software (SPSS Statistics 22; IBM, Armonk, NY, USA) was used to determine statistical significance, which was set at *P* < 0.05.

## 3 RESULTS

**Fig. 2:**
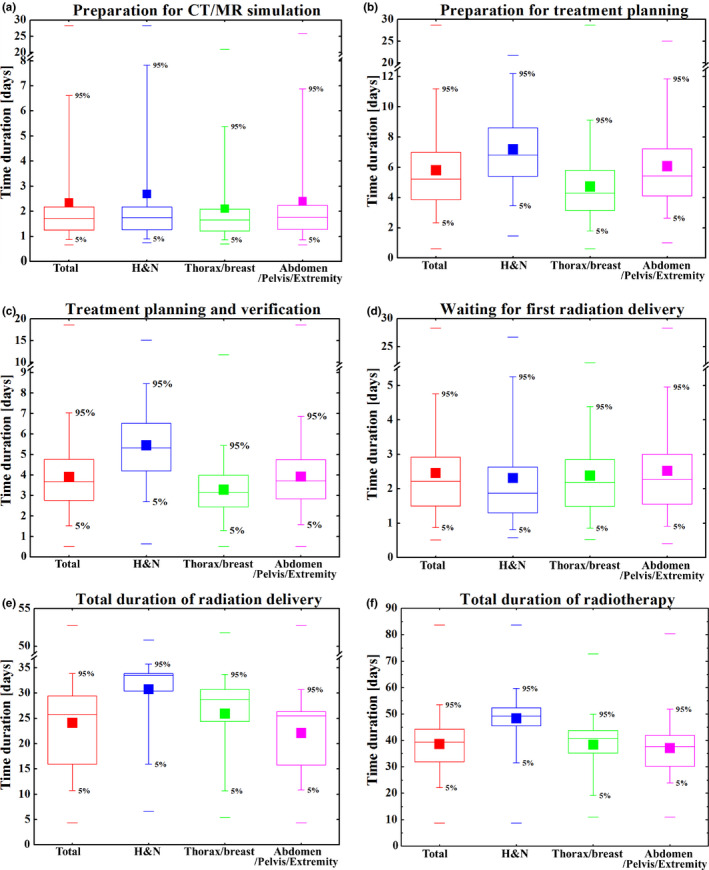
The time durations of different modules and tumor sites, separately. Median with 25% and 75% quartiles (boxes), mean (filled squares), maximum and minimum (lines), and 5% and 95% percentiles (whiskers) are shown.

For the convenience of comparison and statistical analysis, the summarized results were calculated as “days,” with two decimal points (Table 1). Figures 2(a)–2(f) shows the time durations of the modules and tumor sites. The abscissa shows the tumor sites, and the ordinate gives the time duration (in days) for the completing the module. It is noteworthy that this study counted only working days’ times, excluding weekends and legal holidays.

**Table 1:** The time durations of different modules and tumor sites. All data is calculated in days with two decimal places remaining and displayed in the form of mean/median(25%, 75%).

	Total (days)	H&N (days)	Thorax/breast (days)	Abdomen/pelvis/extremity (days)
Preparation for CT/MR simulation	2.24/1.72 (1.24–2.17)	2.74/1.74 (1.27–2.18)	2.11/1.64 (1.20–2.10)	2.31/1.75 (1.26–2.25)
Preparation for treatment planning	5.75/5.22 (3.85–7.02)	7.15/6.82 (5.40–8.61)	4.65/4.27 (3.14–5.81)	5.92/5.43 (4.10–7.22)
Treatment planning and verification	3.88/3.68 (2.77–4.78)	5.40/5.32 (4.20–6.52)	3.22/3.15 (2.46–3.99)	3.81/3.74 (2.83–4.74)
Waiting for first radiation delivery	2.48/2.21 (1.50–2.93)	2.31/1.88 (1.30–2.63)	2.38/2.18 (1.50–2.84)	2.47/2.26 (1.56–3.02)
Total duration of radiation delivery	24.12/25.77 (15.95–29.44)	30.98/33.52 (30.24–33.87)	25.68/28.74 (24.41–30.73)	23.12/25.48 (15.78–26.38)
Total duration of radiotherapy	38.56/39.39 (31.89–44.27)	48.45/49.19 (45.50–52.36)	37.82/40.81 (35.22–43.84)	37.22/37.66 (30.17–41.89)

The times utilized for preparation for computed tomography/magnetic resonance imaging (CT/MR) [Table 1; Fig. 2(a)] based on the tumor site were almost the same, with three‐fourths of all patients spending ca. 1–2 days, except those with “H&N” group, where the average time was slightly longer (nearly 3 days). Fewer than 10% of new patients underwent CT/MR evaluation on the same day they first came to our department.

Overall, the preparation for treatment planning [Table 1; Fig. 2(b)] — including transmission and fusion of CT/MR/PET images, definition of tumor targets and organs at risk, authorization of the contouring, submission of treatment prescriptions — was the most time‐consuming module before irradiation. The median time consumed for this module was 5.22 days. Among the cancer types, the “H&N” group required significantly more time (median time 6.82 days with three‐fourths spending < 9 days) and thoracic/breast group the least time (median time 4.27 days with three‐fourths spending < 6 days). It is worth noting that, although the mean and median times of the “H&N” group were much longer than those of the “Thorax/breast” group and “Abdomen/Pelvis/Extremity” group, the maximum time for the “H&N” group was the shortest (21.72 days), compared with the “Thorax/breast” group (28.64 days) and “Abdomen/Pelvis/Extremity” group (24.99 days), indicating the least deviation in time (20.34 days).

**Fig. 3:**
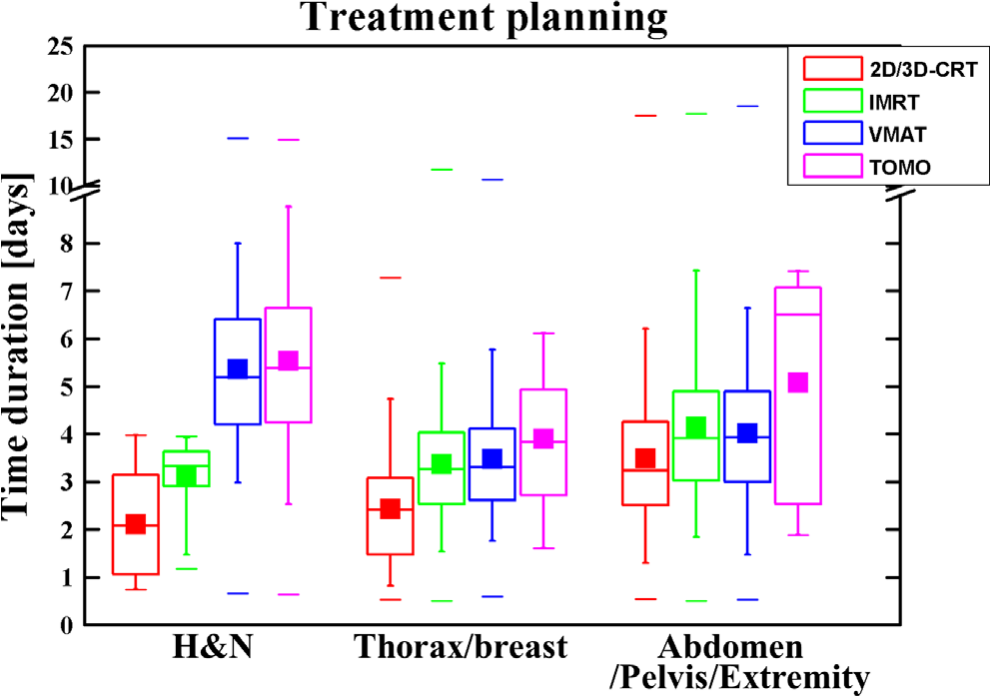
Time utilization for treatment planning using different treatment techniques (2D/3D‐CRT, IMRT, VMAT, and TomoTherapy) of different tumor sites. Median with 25% and 75% quartiles (boxes), mean (filled squares), maximum and minimum (lines), and 5% and 95% percentiles (whiskers) are shown.

The mean and median times consumed for treatment planning and verification [Table 1; Fig. 2(c)]) for all patients were 3.88 and 3.68 days; three‐fourths of all treatment plans were completed in 4.78 days; and 95% (nearly all) plans required <7.04 days. Differences between three groups were significant, with large time variations. The much shorter times for the “thorax/breast” group (mean 3.22 days, median 3.15 days) were most likely the result of relatively simple techniques and routine planning strategies compared with those of the “H&N” group (mean 5.40 days, median 5.32 days). Table 2 and Fig. 3 show the times spent on treatment planning using various treatment techniques [two‐dimensional/three‐dimensional conformal radiotherapy (2D/3D‐CRT), intensity‐modulated radiotherapy (IMRT), volumetric‐modulated arc therapy (VMAT), TomoTherapy (TOMO)], showing the overall trend of increasing time consumption from 2D/3D‐CRT → IMRT→VMAT → TOMO. Among these techniques, the mean and median times consumed with VMAT and TomoTherapy for the “H&N” group were the longest: 5.31 and 5.18 days vs 5.58 and 5.38 days, respectively. It is worth noting that IMRT planning was much shorter than VMAT planning for H&N tumors for the reason that almost all complicated H&N planning was performed using VMAT and TOMO. Only the planning for simple and small target volume tumors utilized IMRT, which took a much shorter time. For the “Thorax/breast” and “Abdomen/Pelvis/Extremity” groups, the times spent using IMRT and VMAT were almost the same. The times for both IMRT and VMAT were longer than the times for 2D/3D CRT and shorter than those for TOMO.

**Table 2:** Time utilization for treatment planning using different treatment techniques (2D/3D‐CRT, IMRT, VMAT, and TomoTherapy) of different tumor sites. All data is calculated in days with two decimal places remaining and displayed in the form of mean/median(25%, 75%).

	2D/3D‐CRT (days)	IMRT (days)	VMAT (days)	Tomotherapy (days)
H&N	2.11/2.09 (1.06–3.14)	3.12/3.33 (2.90–3.63)	5.31/5.18 (4.20–6.41)	5.58/5.38 (4.25–6.65)
Thorax/breast	2.40/2.42 (1.47–3.08)	3.37/3.27 (2.53–4.03)	3.40/3.32 (2.62–4.11)	3.91/3.88 (2.66–4.97)
Abdomen/pelvis/extremity	3.41/3.24 (2.51–4.26)	4.07/3.92 (3.03–4.90)	3.98/3.93 (3.00–4.90)	4.95/6.50 (2.21–7.24)

After treatment planning and verification were completed and authorized, approximately three‐fourths of patients underwent their first irradiation within 3 days [Table 1; Fig. 2(d)]. The mean and median waiting times for H&N tumors were the shortest (2.31 and 1.88 days, respectively) and those for the “Abdomen/Pelvis/Extremity” group the longest (2.47 and 2.26 days, respectively). Statistically, only ca. 8% of patients underwent their first irradiation within 1 day after the treatment plans were completed and approved. More than 10% of patients had to wait for>4 days, which if not necessary, was considered delayed treatment.

The total duration of radiation delivery [Table 1; Fig. 2(e)] clearly showed the difference in our department’s routine treatment prescriptions for the different tumor groups. Conventional fractionation radiotherapy for H&N tumors required 33 deliveries, with mean and median durations of 30.98 and 33.52 days, respectively. Most treatment prescriptions for the “Thorax/breast” group were 25 or 28 fractions, with mean and median times of 25.68 and 28.74 days, respectively. Because of the diversity of tumors of the “Abdomen/Pelvis/Extremity” group and the different treatment prescriptions, conventional fractionation ranged from 25 to 30 deliveries, and hypofractionation with prescription larger than 5 Gy/fraction ranged from 5 to 15 fractions. Hence, the total duration of radiation delivery of the “abdomen/pelvis/extremity” group was the most diverse, with mean and median values of 23.12 and 25.48 days, respectively.

Total duration of radiotherapy [Table 1; Fig. 2(f)] is the total time from establishing medical records in the department to the end of the last radiation delivery (second‐course or multi‐course radiotherapy was beyond the scope of this study). The shortest and longest times for all patients were 8.76 and 83.68 days, respectively. That of the 5% to 95% interval for all patients ranged from 22.16 to 53.52 days, with mean and median times of 38.56 and 39.39 days, respectively. Among the tumor types, the “H&N” group had the longest total radiotherapy duration, with mean and median values of 48.45 and 49.19 days, respectively. “Thorax/breast” group’s total radiotherapy duration was the second longest, with mean and median times of 37.82 and 40.81 days, respectively. The total duration of the “Abdomen/Pelvis/Extremity” group was the shortest, with mean and median times of 37.22 and 37.66 days, respectively. One‐half of patients with “ H&N” group’s tumor had the shortest 25% to 75% interval, with a difference of only 6.85 days; patients with “Thorax/breast” group’s tumor had a difference of 8.63 days; and those with “Abdomen/Pelvis/Extremity” group’s tumor had a difference of 11.71 days.

## 4 DISCUSSION

An important factor affecting the quality of medical care and cancer‐patient satisfaction is mastering the radiotherapy workflow and minimizing the entire treatment time, especially patients’ waiting time. This study assessed the efficiency of the current radiotherapy management in our department by analyzing real‐time clinical time information. Our results could provide a useful research method for other departments. Evaluating the time spent on all the core modules of radiotherapy could help the staff reserve proper working time for each radiotherapy module, reduce unnecessary patients’ waiting time, avoid the work pressure on medical teams, improve medical efficiency, and ensure quality of medical care.

This study analyzed real‐time treatment information over a 3‐yr period in parallel with routine clinical work, thereby avoiding the impact of statistical results with annual work‐cycle fluctuations. The data size of the treatment information of 17 620 patients was this study’s foremost advantage as it is a larger collection than has been reported in previously published studies. The study included all patients who underwent radiotherapy for the first time in our department, maximally preventing repeated evaluations due to multiple treatments of second‐course or multi‐course radiotherapy, which could result in erroneous treatment information.

To maintain consistency and reliability, the study used a unified MOSAIQ 24‐h network clock for collection of real‐time information, with the statistical time accurate to the second. Conventionally, this study calculated data collected only on work days, automatically ignoring weekends and statutory holidays. Compared with previous manual recording methods, use of this automatic radiotherapy management software (MOSAIQ system) improved the accuracy of the study and avoided adding an extra workload on the staff, minimizing any interference between the study tasks and routine clinical work. Also, the use of electronic tools makes it easier to check for anomalous treatment information and correct errors more efficiently. Importantly, the data collection tool used in this study, the MOSAIQ system, is mature commercial software and so makes our study easy to promote and repeat in other departments, which has great practical significance.

### 4.A Impact of current CT/MR scans appointments

The results of the study showed that >90% of patients underwent their first CT/MR scan within three working days after their medical records were established. The difference in waiting time for patients according to their tumor site was small. In the future, therefore, when setting up appointments for routine CT/MR scans, a 3‐day waiting time can be reserved for each patient without distinction, and the patient is informed in advance to avoid disputes. Thus, ca. 10% patients’ treatment time could be shortened. This appointment system can also encourage physicians and technicians to avoid the lack of timely CT/MR scans due to negligence and/or lack of responsibility, which could delay subsequent treatment.

### 4.B Impact of current contouring and ward rounds

The statistical results of the current preparation time for treatment planning revealed the difference in the workload and difficulty of tumor target and organs at risk contouring of different tumor types. They also reflected the work efficiency and responsibility of different physician groups. Generally, contouring the tumor target was performed only by the responsible senior physician, whereas routine contouring of the organs at risk was performed by resident physicians and reviewed by a senior physician. Therefore, differences in the proficiency of resident physicians and the responsibility level of senior physicians would lead to different times spent in this module. In addition, all contouring of the tumor target and organs at risk in patients with different tumor types/sites should be finally authorized during departmental ward rounds before subsequent treatment. Therefore, appropriately arranging the time and frequency of ward rounds is vital for reducing patients’ unnecessary waiting time. In the current study, H&N tumors required the most time for contouring and authorization — approximately 1.5 times longer than that required for thoracic and abdominal tumors. To improve efficiency, our department conducts ward rounds according to the tumor site — that is, twice a week for H&N tumors and once a week for thoracic and abdominal tumors, respectively. In case of emergent patients, temporary ward rounds are performed to reduce patients’ waiting time while ensuring medical safety. As a result, the treatment time of all H&N tumors in regular patients and emergent patients could be shortened, which accounts for ca. 30% of all patients.

### 4.C Impact of current treatment appointments and planning

The former department procedure makes it the planning physicists’ duty to set up an appointment for the treatment accelerator based on the first treatment time for the patient to occur within 5 days ahead of the planning, according to the prescription’s required treatment technology (i.e., 2D/3D‐CRT, IMRT, VMAT, TOMO). There are several main reasons leading to physicists' failure to complete treatment planning on time. (a) If the planning physicist fails to make an appointment for the appropriate accelerator, because of the increasing number of cancer patients and the shortage of accelerator resources, the planning cannot start on time. (b) The planning physicist did not reasonably estimate the difficulty of the treatment plan, so the planning time exceeded the appointed treatment time, making the previous appointment invalid, requiring re‐scheduling. (c) For personal reasons (e.g., a heavy workload, temporary leave, lack of adequate sense of responsibility), the planning physicist fails to complete the treatment plan on time. (d) There is another scenario wherein the treatment planning is completed ahead of the appointment time, so the patient must wait and is not treated until the scheduled appointment time. Each of these situations would result in wasting treatment resources, unnecessarily prolonging the patient’s waiting time and delaying timely treatment.

In view of the abovementioned treatment delays, our department has stipulated new procedures for setting up treatment appointments and planning.

- According to the total number of patients and the throughput capacity of each accelerator, appointments for the first treatment are adjusted in time and allocated more reasonably to the available accelerators, thereby avoiding the situation wherein planning physicists fail to make an appointment for the appropriate accelerator within 5 days.
- The appointed treatment time is adjusted according to the actual completion time established in the treatment plan to ensure that the patient can receive his/her first treatment on the same day, thereby avoiding unnecessary waiting time.

In accordance with the results of this study, our department has developed a new performance evaluation system for time consumption during treatment planning. The new system stipulates that the treatment planning for H&N tumors should be completed within five working days, and for thoracic and abdomen tumors within three working days. The new system requires timely investigation of treatment planning that is not completed on time. Treatment planning time could be shortened for>70% of patients with H&N tumors and 20% of those with thoracic and abdominal tumors, which accounts for>40% of all patients.

These new procedures and performance evaluation system help standardize work efficiency and responsibility of physicians and planning physicists. Also important, they reduce the occurrence of delayed treatment and unnecessary patient waiting time.

The incidence of newly diagnosed cancer has been rising in recent decades. Many studies have shown that cancer is a common disease in the elderly. Thus, with our aging population, the incidence of malignant tumors and the need for treatment will undoubtedly continue to grow. Efficient management of radiotherapy workflow and controlling the treatment time can improve efficiency on the premise of ensuring quality treatment. In total, >60% of cancer patients would benefit from the above proposed modifications, and the total treatment time could be shortened. Subsequent studies will continue to evaluate the overall treatment time of the improved radiotherapy workflow management and assess the clinical outcomes.

## 5 CONCLUSIONS

The present study is part of a project of quality and management control in our radiation oncology department. With the increasing clinical workload, it is particularly important to study daily problems during the radiotherapy process. We found significant differences in the time spent during the radiotherapy core modules depending on the anatomical tumor site, for which the related time control and appointment management had not been modified. The study and data presented here allow dynamic estimation of time consumption for the radiotherapy core procedures at the various tumor sites and regarding which of the most common radiotherapy techniques is used, including 2D/3D‐CRT, IMRT, VMAT, and TOMO. The time control and appointment regulations proposed in this study should help managers make timely, appropriate decisions based on real‐time radiotherapy information, thereby meeting daily clinical needs. Our results could also be considered a standard protocol for future research.

This study accurately recorded the time spent in each radiotherapy core module and established a reliable method for evaluating the entire radiotherapy process. With the MOSAIQ system, we analyzed the treatment data of 17 620 cancer patients during a recent 3‐yr period, summarized the advantages and disadvantages of the previous radiotherapy workflow management, and offered suggestions for improving time control and appointment regulations. This study aimed to provide reliable guidance using exact time consumption during clinical radiotherapy based on accurate treatment time information. To improve the patient's medical experience and satisfaction, the current study pointed out a clear direction for future improvement of radiotherapy workflow management and time control in our radiation oncology department.

## CONFLICT OF INTEREST

The authors report no conflicts of interest with this study. We declare that we do not have any commercial or associative interest that represents a conflict of interest in connection with this work.
